# Clinical relevance of neutrophil-to-lymphocyte ratio and mean platelet volume in pediatric Henoch–Schonlein Purpura: a meta-analysis

**DOI:** 10.1080/21655979.2020.1865607

**Published:** 2021-01-08

**Authors:** Bowen Li, Qian Ren, Jizu Ling, Zhongbin Tao, Xuemei Yang, Yuning Li

**Affiliations:** a Department of Pediatrics, The First Hospital of Lanzhou University, Lanzhou, China; b Department of Gastroenterology, The First Hospital of Lanzhou University, Lanzhou, China

**Keywords:** NLR, MPV, Henoch-Schonlein Purpura, gastrointestinal involvements

## Abstract

The association of neutrophil-to-lymphocyte ratio (NLR) and mean platelet volume (MPV) with the severe gastrointestinal (GI) involvement in pediatric Henoch–Schonlein Purpura (HSP) has been reported in many studies. However, the conclusions from the previous studies were controversial. Therefore, for the first time, we performed a meta-analysis to systematically evaluate the relationship of NLR and MPV to the severe GI involvements. We retrieved PubMed, EMBASE, Web of Science, and Chinese National Knowledge Infrastructure (CNKI) (up to October 2020) thoroughly to acquire eligible studies. The pooled standard mean difference (SMD) with 95% confidence interval (CI) was used to describe the correlation of NLR and MPV with the severe GI involvement. A total of 1^2^ studies comprising 2168 patients with HSP were included in this meta-analysis. Our combined analysis showed that NLR in HSP patients with the severe GI involvement was significantly higher than that in those without the severe GI involvement (SMD = 1.37; 95% CI: 0.70–2.05; p < 0.01). In addition, a lower MPV was observed in children with severe GI involvement (SMD = −0.29; 95% CI: −0.56 – −0.01, p = 0.042). Our sensitivity analysis and publication bias evaluation indicated that our combined results were reliable. Taken together, our study suggested NLR and MPV may be used as biomarkers for predicting or diagnosing the severe GI involvement in children with HSP. Nevertheless, more homogeneous studies with a larger sample size are required to validate these findings.

## Graphical abstract

**Figure:**
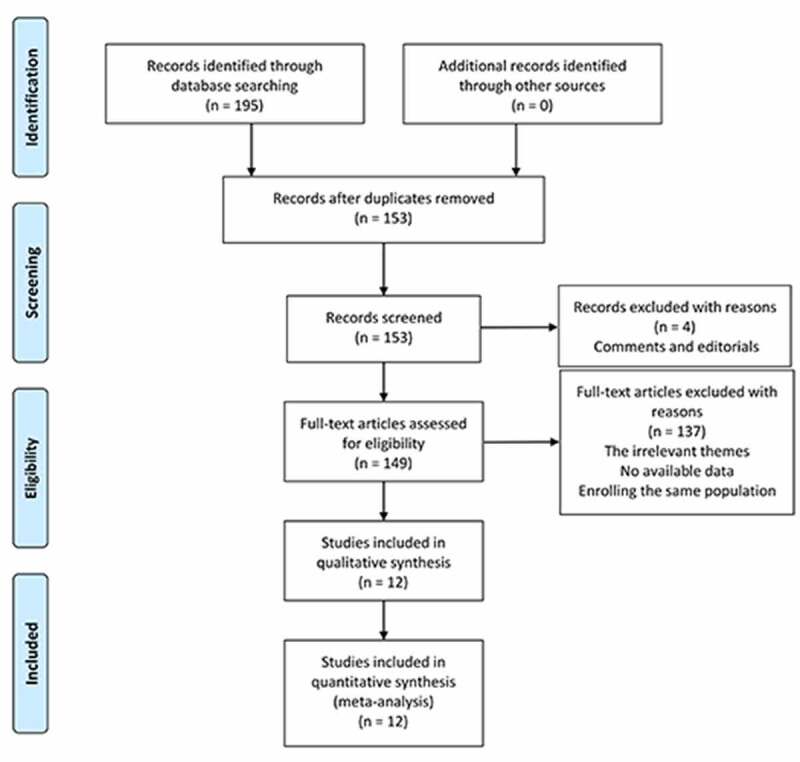

## Introduction

Henoch-Schönlein Purpura (HSP) also named immunoglobulin A (IgA) vasculitis, is the most common systemic vasculitis in childhood [1,2]. The clinical manifestations of HSP includes the palpable purpura and multiple organ involvements, such as gastrointestinal damage, arthritis, and nephritis [3]. HSP has been considered to be a typically self-limited disease and most patients usually have a favorable prognosis when only supportive care is provided [4]. However, the severe gastrointestinal (GI) involvements, such as GI bleeding, bowel edema, intussusception, and intestinal perforation, may increase the morbidity and mortality in children with HSP [5,6]. In addition to supportive care, HSP patients with severe GI involvements should be treated with more positive strategies including fasting, parenteral nutrition, high-dose corticosteroid, and even surgical intervention [7,8]. The clinical manifestations of the severe GI involvements tend to be insidious and atypical in children with HSP [6], which always prevents physicians from timely evaluating the disease severity and providing proper treatment. Therefore, it is very important to develop the practical and economical biomarkers for precisely predicting and rapidly diagnosing the severe GI involvements in children with HSP.

The autoimmune inflammatory response has been considered to be involved in the development of HSP, although the pathogenesis of HSP remains incompletely elucidated. Several pro-inflammatory cytokines, such as tumor necrosis factor (TNF)-α, interleukin (IL)-1, IL-4, IL-6, and IL-8 may contribute to the pathogenesis of HSP [9–12]. Additionally, IgA deposits in small vessels, as the main pathological feature of HSP, could not only lead to complement activation but also increase the neutrophil infiltration in the wall of the involved vessels [13,14]. Furthermore, neutrophil-dominant inflammation plays an essential role in the development of HSP [14]. Consistently, increasing evidence suggested that some neutrophil-associated laboratory markers may serve as biomarkers for the GI involvements in HSP patients. Blood neutrophil-to-lymphocyte ratio (NLR) is a systemic inflammatory marker that can be easily obtained from blood routine examination. Several studies showed NLR was higher in HSP patients versus healthy controls and correlated with the presence of severe GI involvements, implying that NLR may be used as a beneficial biomarker for predicting or diagnosing the severe GI involvements [6,14,15]. However, a most recent study reported that there was no significant relationship between NLR and the severe GI involvements in children with HSP [5]. Overall, the conclusions about the association of NLR with the severe GI involvements in children with HSP remains conflicting and further studies are required to resolve this controversy.

Mean platelet volume (MPV) also is an easily accessible and inexpensive laboratory marker that reflects platelet function and activation [16]. Interestingly, MPV has been considered to be an inflammatory biomarker [17] and a lower MPV may reflect the activity and severity of multiple inflammatory diseases [18–20]. For example, a reduced MPV positively correlated with active phase in gastrointestinal tract inflammatory diseases, such as ulcerative colitis and Crohn’s disease [21,22]. Furthermore, accumulating evidence shows that there is a close relationship between a lower MPV and the severe GI involvements in patients with HSP [15,23,24], indicating MPV may also serve as a biomarker for diagnosing or predicting the severe GI involvements. Nevertheless, some studies reported MPV was not associated with severe GI involvements in patients with HSP [14,25,26]. Obviously, there is no consensus about the association of MPV with the severe GI involvements in children with HSP.

The association of NLR and MPV with the severe GI involvements in children with HSP remains controversial [6,14,15,23,25,27–29] and the sample sizes in those previous studies were small, so in this study, we performed a meta-analysis of the current literature to determine whether NLR and MPV correlates with the severe GI involvements in children with HSP.

## Method

### Literature search

A comprehensive literature search was performed in three databases including PubMed, EMBASE, Web of Science, and Chinese National Knowledge Infrastructure (CNKI) (up to October 2020). The terms used for literature search included Henoch-schönlein purpura, HSP, immunoglobulin A vasculitis, IgA vasculitis, mean platelet volume, MPV, neutrophil to lymphocyte ratio, and NLR. The search strategy in PubMed was as following (‘Henoch schönlein purpura’[Title/Abstract] OR ‘HSP’[Title/Abstract] OR ‘immunoglobulin a vasculitis’[Title/Abstract] OR ‘IgA vasculitis’[Title/Abstract]) AND (‘mean platelet volume’[Title/Abstract] OR ‘MPV’[Title/Abstract] OR (‘neutrophil to lymphocyte ratio’[Title/Abstract] OR ‘NLR’[Title/Abstract])).

### Study selection

The inclusion criteria included: (1) cross-sectional, case-control or cohort studies; (2) the studies enrolled pediatric patients diagnosed with HSP; (3) the data of NLR or MPV in HSP patients with and without the severe GI involvements could be extracted; (4) the severe GI involvements were defined as colicky abdominal pain, bowel edema in ultrasonography or GI bleeding.

The exclusion criteria were as follows: (1) if the same population was enrolled in two or more studies, the one with a larger sample size was included; (2) the studies were published as reviews, editorials, conference abstracts, case reports, and noncomparative studies; (3) the full text was unavailable; (4) the data of NLR or MPV in HSP patients with and without the severe GI involvements could not be extracted; (5) the studies have focussed on the other topics.

### Data extraction and quality assessment

All the data were independently extracted by two independent investigators. Any disagreement was resolved by the third investigator. The extracted data included the name of the first author, publication year, recruitment period, country, the mean age of HSP patients, girls’ ratio of patients, case number, the definition of GI involvements, the mean value with standard deviation (SD) of MPV and NLR. The Newcastle–Ottawa scale (NOS) was applied to evaluate the quality of the included studies. In the NOS system, each study could be assigned with a score of 0–9 points and scores ≥6 suggest that studies are high-quality.

### Statistical analysis

The pooled analysis of data was performed using Stata SE12.0 (StataCorp, College Station, TX). Standard mean difference (SMD) with 95% CI was used to analyze the continuous variables. SMD > 0 coupled with 95% CI not crossing 0 suggested that NLR or MPV were significantly higher in HSP patients with severe GI involvements. Inversely, SMD < 0 coupled with 95% CI not crossing 0 indicated that NLR or MPV was significantly lower in HSP patients with severe GI involvements. The Q statistic and I^2^ was used to test and quantify heterogeneity. The random effect model is chosen to calculate the pooled SMD with 95% CI, when the *p* value of the Cochrane Q test was <0.05 or I^2^ is ≥50% [30,31]. Otherwise, the random effect model was used. Sensitivity analyses were performed by omitting a single study in each step [30,31]. Subgroup analyses were conducted by age, country, and the definition of severe GI involvements.

Sensitivity analyses were performed to confirm the stability of the pooled effects by omitting the included studies one by one. The Begg funnel plot and Egger’s test were used to evaluate the publication bias [32,33]. According to the guidance from the Cochrane handbook, publication bias assessment should be performed only when there are at least 10 eligible studies for the outcome measures.

## Results

### Study search and selection

**Figure 1.**
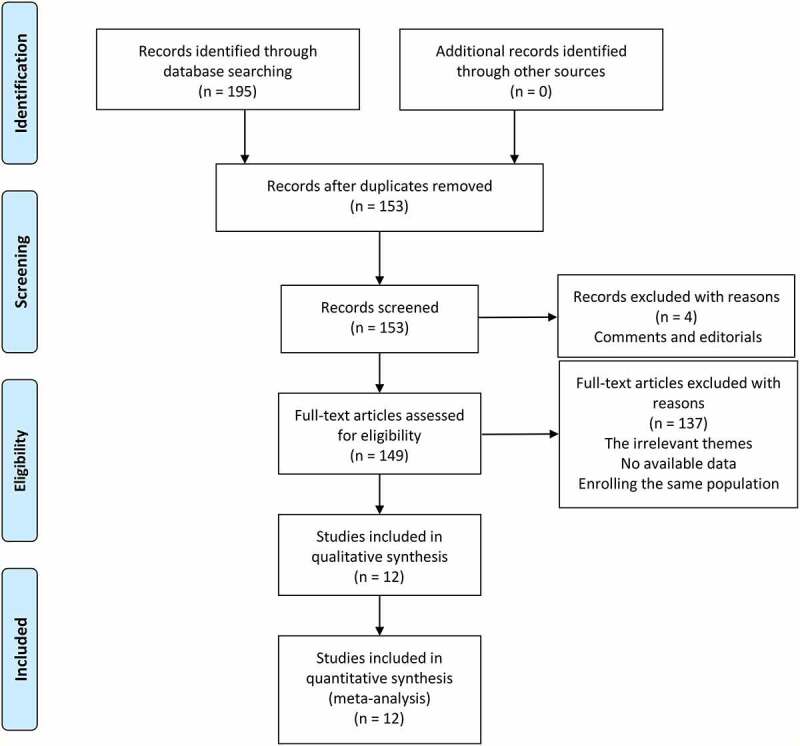
Flow chart of literature search and selection

We searched 195 potential studies in PubMed, Web of Science, and China National Knowledge Infrastructure (CNKI) databases. After removing 42 duplicated records, 153 studies were further screened by titles and abstracts. In this procedure, four studies were excluded due to comments and editorials. Then, we made a careful review of the remaining studies and 137 studies were removed due to the other themes, insufficient data, or the enrollment of the same population. Finally, 12 studies comprising 2168 patients with HSP were included for this meta-analysis [5,6,14,15,25–29,34–36]. The flow chart diagram of the study selection is illustrated in Figure 1.

### Study characteristics

The included studies were published from 2013 to 2020. The sample sizes of the eligible studies ranged from 49 to 524. Among the included studies, five studies were performed in China and analyzed the Chinese population [25,28,29,34,36]; only one study was conducted in Korea and analyzed the Korean population [6], and six studies were performed in Turkey and enrolled the Turkish population [5,14,15,26,27,35]. A total of 10 studies investigated the association between MPV and the severe GI involvements in patients with HSP [5,6,14,15,25,26,28,29,35,36] and 8 studies referred to the relationship between NLR and the severe GI involvements [5,6,14,15,26,27,34,36]. The severe GI involvement was defined as the GI bleeding in 10 eligible studies, whereas the severe colicky abdominal pain, bowel edema, GI bleeding, and intussusception were regarded as the severe GI involvements in two studies [5,6,14,15,25,26,28,29,35,36]. The quality scores of the included studies ranged from 6 to 7. The information about the study characteristics is summarized in Table 1.

**Table 1.** The basic characteristics of the included studies

Study	Recruitment duration	Country	Age	Sample size	Girls (%)	Definition of gastrointestinal involvement	Medication prior to blood sampling	Infections	NOS score
			(years)						
Benzer et al. [35]	2008–2013	Turkey	6.8 ± 3.02	75	49.37	GI bleeding	NR	NR	6
Ekinci et al. [14]	NR	Turkey	7.6 ± 3.1	214	43.5	Severe colicky abdominal pain/bowel edema in ultrasonography/overt GI bleeding	No	NR	6
Gayret et al. [27]	2013–2016	Turkey	7.82 ± 3.01	119	37	GI bleeding	No	NR	6
Hong et al [6].	2005–2017	Korea	6.6 ± 2.8	141	44.7	GI bleeding	No	No	7
Liao et al. [34]	2015–2017	China	7.62 ± 3.44	121	38	GI bleeding	NR	NR	6
Karadağ et al. [5]	2016–2019	Turkey	7.5 ± 3.5	376	47.1	Abdominal pain/vomiting/hematemesis/melena/massive GI bleeding/intussusception	NR	NR	6
Makay et al. [15]	2006–2013	Turkey	6.5 ± 2.6	63	47.6	GI bleeding	NR	NR	6
Sun et al. [25]	2010–2012	China	7.6	137	40.1	GI bleeding	NR	NR	6
Yakut et al. [26]	2015–2018	Turkey	7 ± 3.6	49	42.3	GI bleeding	NR	NR	6
Wang et al. [28]	2012–2014	China	6.85 ± 2.65	524	40.5	GI bleeding	NR	NR	6
Zhai et al. [36]	2014–2016	China	7.7 ± 2.8	148	45.3	GI bleeding	No	No	7
Zhang et al. [29]	2011–2017	China	NR	201	NR	GI bleeding	NR	NR	6

Abbreviation: GI: Gastrointestinal; NR: not reported; NOS: Newcastle-Ottawa scale.

### Association of MPV and NLR with severe GI involvements in HSP

**Figure 2.**
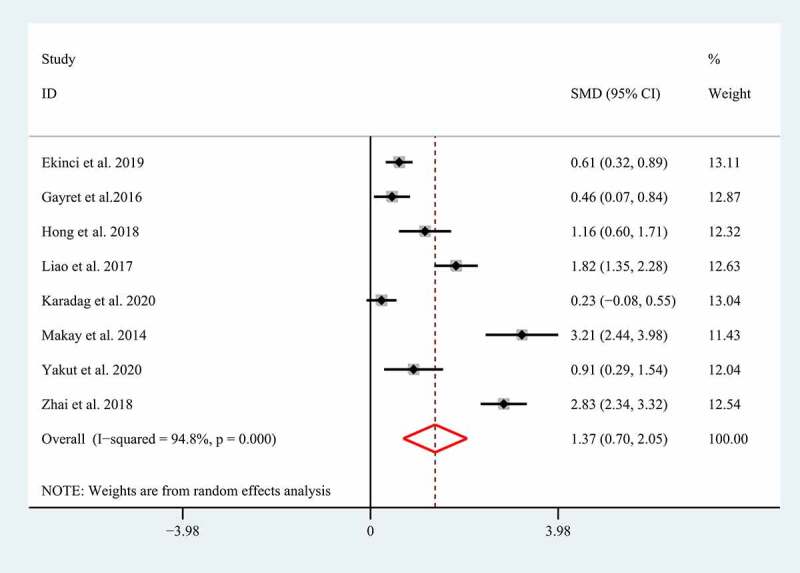
Meta-analysis of eight studies reporting the association between NLR and the GI involvement in HSP patients. SMD: standard mean difference; NLR: neutrophil-to-lymphocyte ratio; HSP: Henoch-Schonlein Purpura; GI: gastrointestinal

Severe GI involvements such as GI bleeding, bowel edema, intussusception, and intestinal perforation have been supposed to increase the morbidity and mortality in children with HSP [5,6], so HSP patients with severe GI involvements should be treated with a positive therapeutic strategy including routine supportive care, fasting, parenteral nutrition, high-dose corticosteroid, and even surgical intervention [7,8]. Unfortunately, the clinical manifestations of severe GI involvements always tend to be insidious and atypical [6], which always results in a delayed diagnosis and treatment. Growing studies showed NLR and MPV correlated with the severe GI involvements in children with HSP, suggesting they may be used as promising biomarkers for diagnosing the severe GI involvements. However, the conclusions regarding the association of NLR and MPV with the severe GI involvements in children with HSP remain controversial [6,14,15,23,25,27–29]. Therefore, herein we performed a meta-analysis of the current literature to determine whether NLR and MPV correlates with severe GI involvements in children with HSP. A total of eight eligible studies with 1231 patients with HSP explored the association between NLR and severe GI involvements [5,6,14,15,26,27,34,36]. The random-effect model was selected to conduct the combined analysis of the association between NLR and the severe GI involvements due to the significant heterogeneity (I^2^ = 94.8%; p < 0.01). The result suggested that a higher NLR correlated with the severe GI involvement in patients with HSP (SMD = 1.37; 95% CI: 0.70–2.05; p < 0.01) (Figure 2).

**Figure 3.**
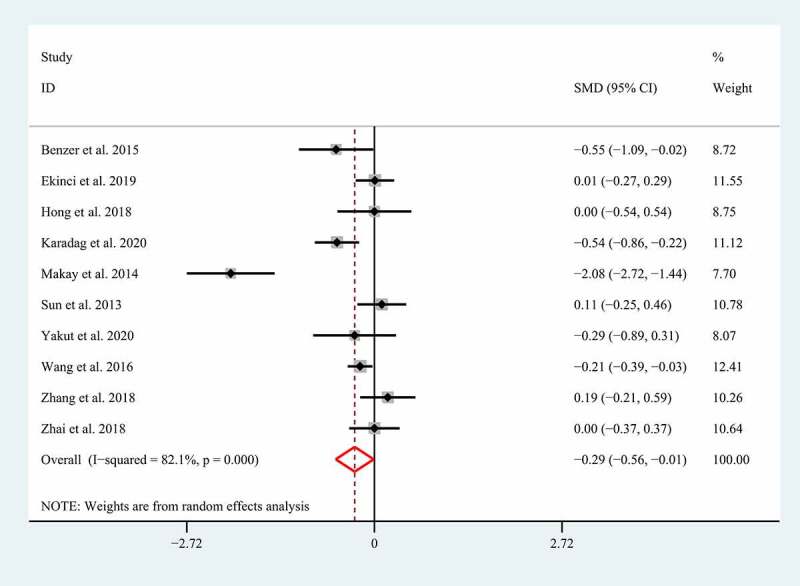
Meta-analysis of 10 studies reporting the association between MPV and the GI involvement in HSP patients. SMD: standard mean difference; MPV: mean platelet volume; HSP: Henoch-Schonlein Purpura; GI: gastrointestinal

The association between MPV and the severe GI involvement was reported in 10 studies with 1928 HSP patients [5,6,14,15,25,26,28,29,35,36]. In view of the significant heterogeneity (I^2^ = 82.1%; p < 0.01), we performed a meta-analysis of the association between MPV and the severe GI involvement using the random effect model. Although most studies suggested that MPV was not related to the severe GI involvement in HSP, our meta-analysis found an adverse relationship between MPV and the severe GI involvement in patients with HSP (SMD = −0.29; 95% CI: −0.56 – −0.01, p = 0.042) (Figure 3).

### Sensitivity analysis and subgroup analysis

**Figure 4.**
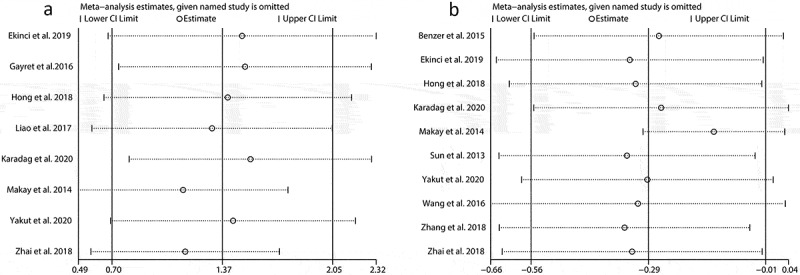
Sensitivity analysis for NLR (a) and MPV (b). SMD: standard mean difference. NLR: neutrophil-to-lymphocyte ratio; MPV: mean platelet volume

Because there was significant heterogeneity in our combined results, we performed the sensitivity analysis and subgroup analysis to minimize the latent confounding factors for our pooled results, and to identify what factors mainly accounted for the heterogeneity. Sensitivity analyses were performed by consecutively omitting a single study. The pooled effects of the association between NLR and the severe GI involvement did not alter significantly when any individual study was excluded (Figure 4(a) and Supplement 1), indicating any single study could not explain the heterogeneity for NLR. Similarly, no matter which study was excluded, the pooled effects still supported that a lower MPV was related to the presence of the severe GI involvement (Figure 4(b) and Supplement 2). However, deletion of the study by Makay et al. significantly altered the pooled effect of the association between MPV and the severe GI involvement, implying the study by Makay et al. might partly account for the heterogeneity for MPV.

The subgroup analyses were performed by age, country, and the definition of the severe GI involvement. The results showed the substantial heterogeneity still existed in all the subgroups for NLR (Table 2) and MPV (Table 3), which indicated these factors might not explain the heterogeneity. Additionally, we found that NLR was positive with the severe GI involvement regardless of age, country, and the definition of severe GI involvement (Table 2 and Supplement 3–5). However, no close correlation between MPV and severe GI involvement was identified in most subgroups, except for Turkey population group (SMD = −0.66; 95% CI: −1.24 – −0.07, p = 0.03) (Table 3 and Supplement 6–8). The exact reasons for this inconsistency are rather complex and one of the explanation may be that the sample sizes in subgroup analyses were small, which could lead to a false-negative result. Therefore, more longitudinal studies with larger sample sizes are required to further confirm whether MPV is associated with severe GI involvement in children with HSP.

**Table 2.** Subgroup analysis for the association between NLR and the severe GI involvement in HSP

Subgroup	Study numbers	SMD (95 CI%)	*p*-value	Heterogeneity	
				I^2^ (%)	*p*-value
**1. Age**					
< 7 years	2	2.17 (0.15–4.18)	0.04	94.5	< 0.01
≥ 7 years	6	1.13 (0.40–1.86)	< 0.01	95.0	< 0.01
Combined	8	1.37 (0.70–2.05)	< 0.01	94.8	< 0.01
**2. Country**					
China	2	2.32 (1.33–3.31)	< 0.01	92.1	< 0.01
Korea	1	1.16 (0.60–1.71)	< 0.01	-	-
Turkey	5	1.00 (0.34–1.66)	< 0.01	93.3	< 0.01
Combined	9	1.37 (0.70–2.05)	< 0.01	94.8	< 0.01
**3. Definition of severe GI involvement**					
GI bleeding	6	1.71 (0.85–2.58)	< 0.01	93.9	< 0.01
Other	2	0.43 (0.06–0.80)	0.02	66.3	0.09
Combined	8	1.37 (0.70–2.05)	< 0.01	94.8	< 0.01

Abbreviation: GI: Gastrointestinal; SMD: standard mean difference. NLR: neutrophil-to-lymphocyte ratio; HSP: Henoch-Schonlein Purpura.

**Table 3.** Subgroup analysis for the association between MPV and the severe GI involvement in HSP

Subgroup	Study numbers	SMD (95 CI%)	*p*-value	Heterogeneity	
				I^2^ (%)	*p*-value
**1. Age**					
< 7 years	3	−0.87 (−2.02–0.29)	0.14	92	< 0.01
≥ 7 years	6	−0.15 (−0.33–0.04)	0.11	51.0	0.07
Combined	9	−0.34 (−0.64 – −0.05)	0.02	82.7	< 0.01
**2. Country**					
China	4	−0.03 (−0.23–0.17)	0.78	40.0	0.17
Korea	1	0.00 (−0.54–0.54)	1	-	-
Turkey	5	−0.66 (−1.24 – −0.07)	0.03	88.9	< 0.01
Combined	10	−0.29 (−0.56 – −0.01)	0.04	82.1	< 0.01
**3. Definition of severe GI involvement**					
GI bleeding	8	−0.30 (−0.66–0.05)	0.09	83.9	< 0.01
Other	2	−0.26 (−0.80–0.28)	0.34	84.4	0.01
Combined	10	−0.29 (−0.56 – −0.01)	0.04	82.1	< 0.01

Abbreviation: GI: Gastrointestinal; SMD: standard mean difference; MPV: mean platelet volume; HSP: Henoch-Schonlein Purpura.

### Publication bias

**Figure 5.**
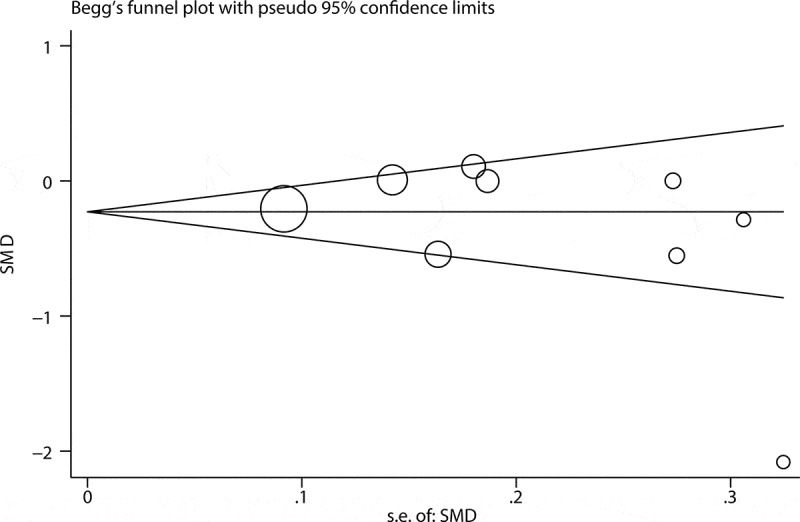
Funnel plot describing the publication bias of MPV. SE: standard error. SMD: standard mean difference; MPV: mean platelet volume

Begg funnel plot and Egger’s test was used to evaluate publication bias for MPV. The results showed Begg funnel plot was symmetrical (Figure 5) and the P-value of Egger’s test was less than 0.05 (p = 0.38) (Supplement 9), indicating no significant publication bias for MPV. Based on the Cochrane handbook, it is proper to conduct publication bias assessment only when there are no less than 10 eligible studies for the outcome measures. Therefore, we did not assess publication bias for NLR owing to less than 10 studies reporting the association of NLR with severe GI involvements.

## Discussion

IgA1 deposits in small vessels are the major pathological feature of HSP and could promote the recruitment of neutrophils into the involved small vessels [13,14], indicating the importance of neutrophil-dominant inflammation in the development of HSP. In addition, the acute phase of HSP is always accompanied by increased release of the proinflammatory cytokines [9–12]. Therefore, the abnormal inflammation may play a key role in the pathogenesis of HSP and specific inflammatory biomarkers may be used to predict or diagnose severe GI involvement. NLR, an easily accessible laboratory marker, has been considered to be a systemic inflammation indicator in various diseases. For example, NLR was markedly increased in patients with systemic lupus erythematosus (SLE) versus healthy control and might predict more active disease status and lupus nephritis [37]. In addition, NLR was demonstrated to correlate with disease activity in patients with rheumatoid arthritis [38]. Similarly, Makay et al. first reported that NLR was increased in children with HSP compared to healthy controls and it might predict the GI bleeding [24]. Afterward, several studies further validated the correlation between NLR and the GI bleeding in HSP [36,39,40], suggesting implying that NLR may be used as a beneficial biomarker for predicting or diagnosing severe GI involvements. However, a few studies suggested that there was no association between NLR and GI involvement in patients with HSP.

Considering the conflicting results, in this study we performed a meta-analysis to further explore this issue. Our data showed that NLR was increased in HSP children with severe GI involvement compared to those patients without severe GI involvements, suggesting NLR may serve as a promising biomarker to predict or diagnose the severe GI involvement in children with HSP.

MPV is also an easily accessible and inexpensive laboratory marker reflecting platelet function and activation [16]. Multiple proinflammatory cytokines can lead to platelet activation in inflammatory diseases, during which an abundance of small-sized platelets are released into the peripheral blood, consequently decreasing MPV value [17]. Additionally, large platelets are easily consumed in the scenario of severe inflammatory response compared to small platelets [41]. These previous studies suggested that a lower MPV may reflect the aberrant inflammatory response. In line with this, evidence shows that MPV significantly correlates with the activity and severity of multiple inflammatory diseases [18–20]. For instance, a lower MPV was positively associated with the active phase in gastrointestinal tract inflammatory diseases, such as ulcerative colitis and Crohn’s disease [21,22]. In particular, it was reported that reduced MPV correlated with the severe GI involvement in patients with HSP [15,23,24]. Nevertheless, the other studies found no relationship between MPV and the severe GI involvement in patients with HSP. Considering that the sample sizes might be limited in the previous studies, in the present study we performed a meta-analysis to further evaluate the correlation between MPV and the severe GI involvement in patients with HSP. Our overall-combined analysis showed MPV was lower in HSP children with severe GI involvement. However, most of the subgroup analyses found no close association between MPV and severe GI involvement. The exact reasons for this inconsistency are rather complex and one of the explanation may be that the sample sizes in subgroups were small, which could lead to a false-negative result. Therefore, more longitudinal studies with larger sample sizes are required to further confirm whether MPV is associated with severe GI involvement in children with HSP.

Several limitations in our meta-analysis should be taken into consideration. First, significant heterogeneity was present across the included studies, which may lead to biased results. The differences in many baseline characteristics, such as the morbidities, disease duration, the sampling timing, the therapeutic history before sampling, and the measurement system of NLR and MPV may account for the heterogeneity. However, we could not determine whether these potential confounding factors surely cause heterogeneity due to the unavailable information. Second, HSP patients in the included studies were from China, Turkey, and Korea, so our conclusions may not be generalized to the other ethnic populations. Third, the sample sizes in our subgroup analyses were small and this may lead to false-negative results. At last but not least, although we retrieved several basic databases to identify eligible studies in English and Chinese, some eligible studies published in the other languages were excluded for the incomplete literature search, which may limit the reliability of our combined analysis.

## Conclusion

Taken together, our study showed a higher NLR and lower MPV were significantly correlated with the presence of the severe GI involvement in children with HSP, suggesting that they may be used as the biomarkers for predicting or diagnosing the severe GI involvement. Nevertheless, more homogeneous studies with larger sample sizes are required to validate these findings due to the limitations in our meta-analysis.

## Supplementary Material

Supplemental Material

## Highlight

High NLR correlates with severe GI involvement in pediatric HSP.

High MPV correlates with severe GI involvement in pediatric HSP.

High NLR is related to GI bleeding in pediatric HSP.

High MPV is related to GI bleeding in pediatric HSP.
