# Supplementary material for: Clinical relevance of neutrophil-to-lymphocyte ratio and mean platelet volume in pediatric Henoch–Schonlein Purpura: a meta-analysis
Source: Bioengineered. 2021 Jan 8;12(1):286–95. doi: 10.1080/21655979.2020.1865607 (PMC8291875; doi:10.1080/21655979.2020.1865607)
Supplement: Supplemental Material [file KBIE_A_1865607_SM3228.zip › supplement/Supplement 1.docx]

| Supplement 1: The sensitive analysis for the association between NLR and the severe GI involvement. | | | |
| --- | --- | --- | --- |
| Study omitted | Estimate | [95% Conf. Interval] | |
| Ekinci et al. 2019 | 1.49 | 0.67 | 2.32 |
| Gayret et al.2016 | 1.51 | 0.74 | 2.29 |
| Hong et al. 2018 | 1.41 | 0.65 | 2.17 |
| Liao et al. 2017 | 1.31 | 0.57 | 2.05 |
| Karadag et al. 2020 | 1.55 | 0.80 | 2.29 |
| Makay et al. 2014 | 1.13 | 0.49 | 1.78 |
| Yakut et al. 2020 | 1.44 | 0.69 | 2.19 |
| Zhai et al. 2018 | 1.15 | 0.57 | 1.72 |
| Combined | 1.37 | 0.70 | 2.05 |

Abbreviation: GI: Gastrointestinal; NLR: neutrophil-to-lymphocyte ratio.
