# Supplementary material for: Clinical relevance of neutrophil-to-lymphocyte ratio and mean platelet volume in pediatric Henoch–Schonlein Purpura: a meta-analysis
Source: Bioengineered. 2021 Jan 8;12(1):286–95. doi: 10.1080/21655979.2020.1865607 (PMC8291875; doi:10.1080/21655979.2020.1865607)
Supplement: Supplemental Material [file KBIE_A_1865607_SM3228.zip › supplement/Supplement 2.docx]

| Supplement 2: The sensitive analysis for the association between MPV and the severe GI involvement. | | | |
| --- | --- | --- | --- |
| Study omitted | Estimate | [95% Conf. Interval] | |
| Benzer et al. 2015 | -0.26 | -0.55 | 0.03 |
| Ekinci et al. 2019 | -0.33 | -0.64 | -0.02 |
| Hong et al. 2018 | -0.32 | -0.61 | -0.02 |
| Karadag et al. 2020 | -0.26 | -0.55 | 0.04 |
| Makay et al. 2014 | -0.13 | -0.30 | 0.03 |
| Sun et al. 2013 | -0.34 | -0.64 | -0.04 |
| Yakut et al. 2020 | -0.29 | -0.58 | 0.01 |
| Wang et al. 2016 | -0.31 | -0.66 | 0.04 |
| Zhang et al. 2018 | -0.34 | -0.63 | -0.05 |
| Zhai et al. 2018 | -0.32 | -0.63 | -0.02 |
| Combined | -0.29 | -0.56 | -0.01 |

Abbreviation: GI: Gastrointestinal; MPV: mean platelet volume.
