# Supplementary figures and images for: Clinical relevance of neutrophil-to-lymphocyte ratio and mean platelet volume in pediatric Henoch–Schonlein Purpura: a meta-analysis
Source: Bioengineered. 2021 Jan 8;12(1):286–95. doi: 10.1080/21655979.2020.1865607 (PMC8291875; doi:10.1080/21655979.2020.1865607)

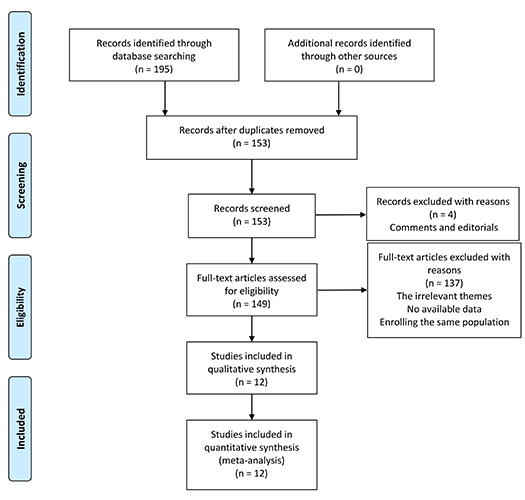

Supplement: Supplemental Material [file KBIE_A_1865607_SM3228.zip › supplement/Graphical abstract.tif]

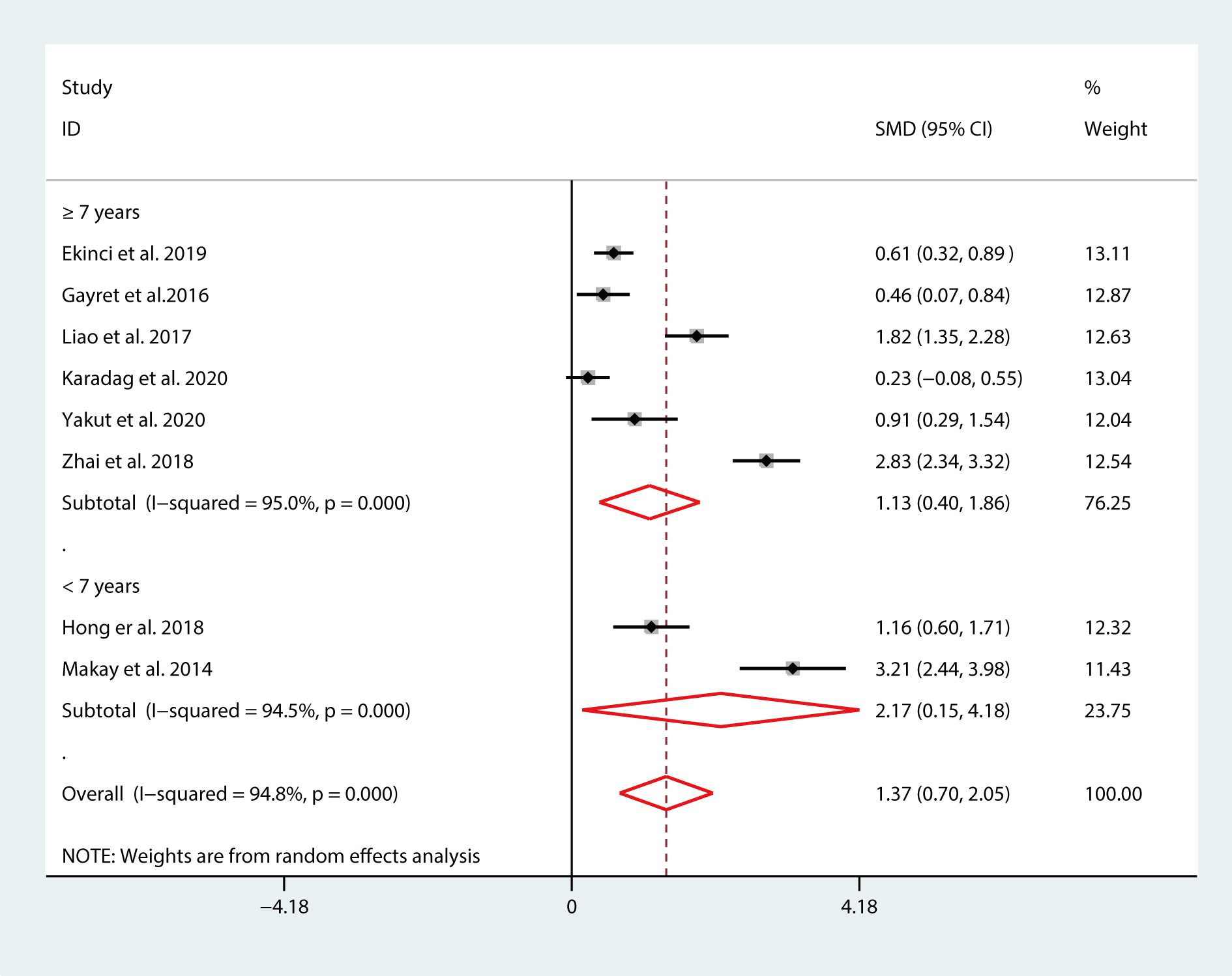

Supplement: Supplemental Material [file KBIE_A_1865607_SM3228.zip › supplement/Supplement 3.tif]

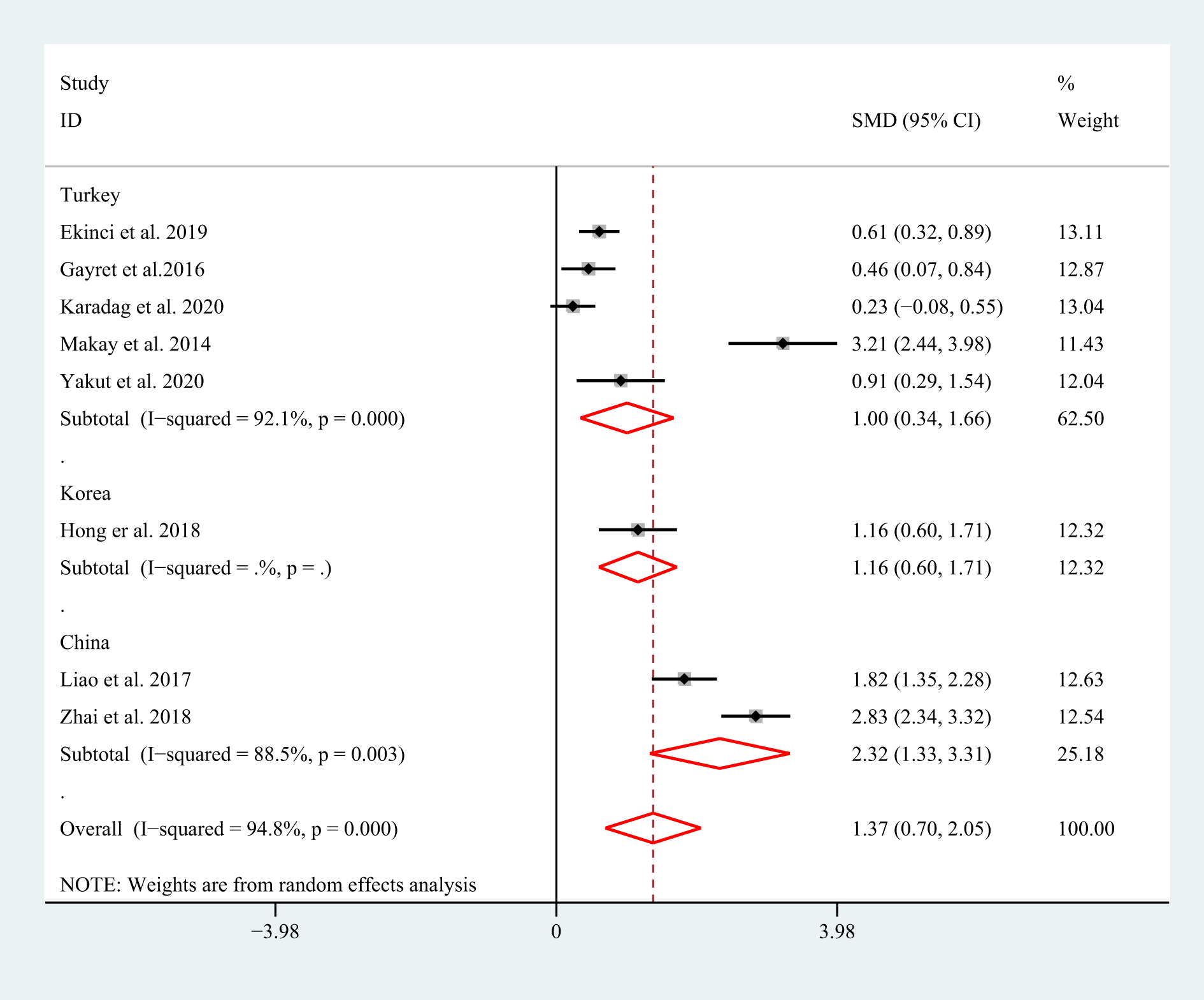

Supplement: Supplemental Material [file KBIE_A_1865607_SM3228.zip › supplement/Supplement 4.tif]

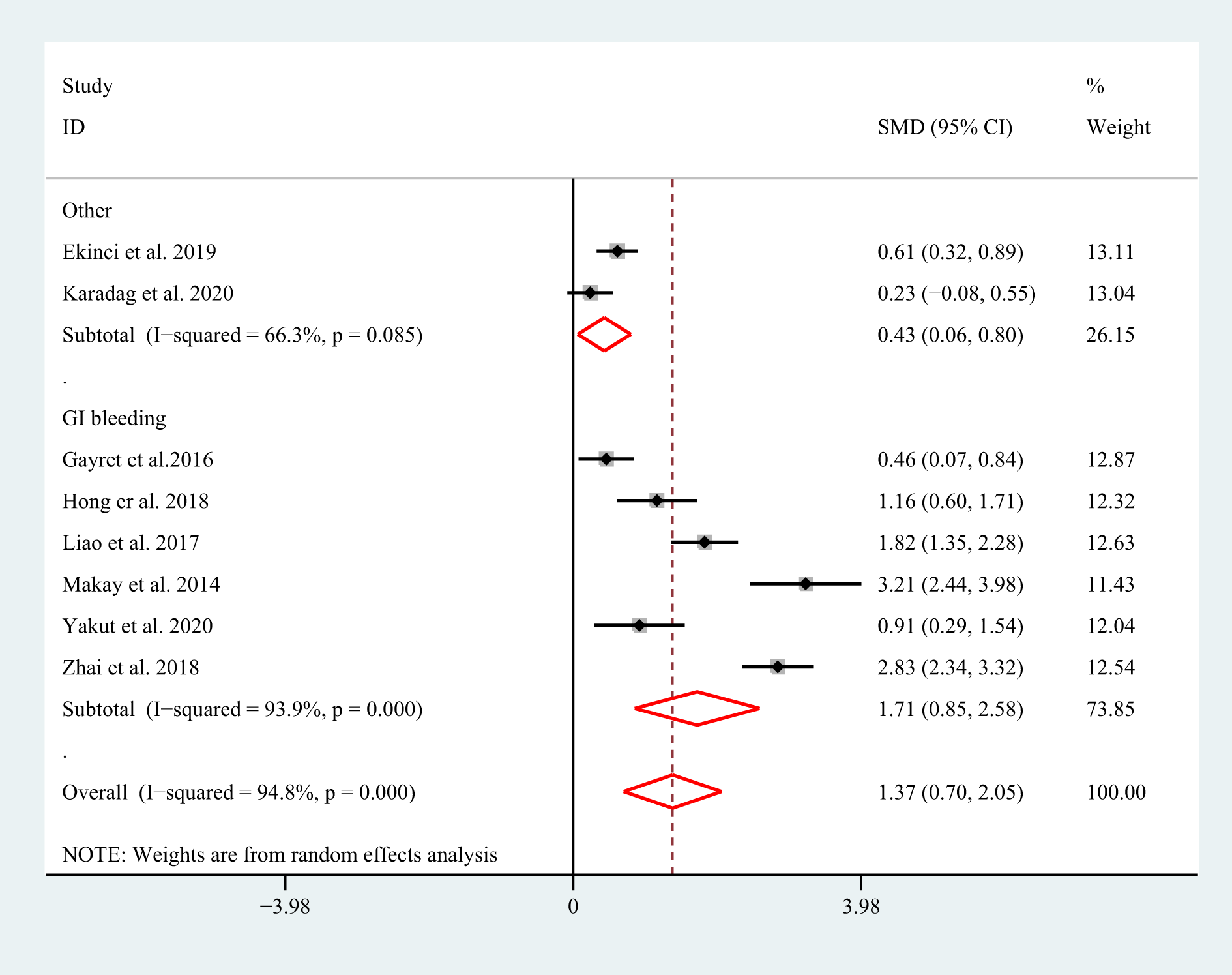

Supplement: Supplemental Material [file KBIE_A_1865607_SM3228.zip › supplement/Supplement 5.tif]

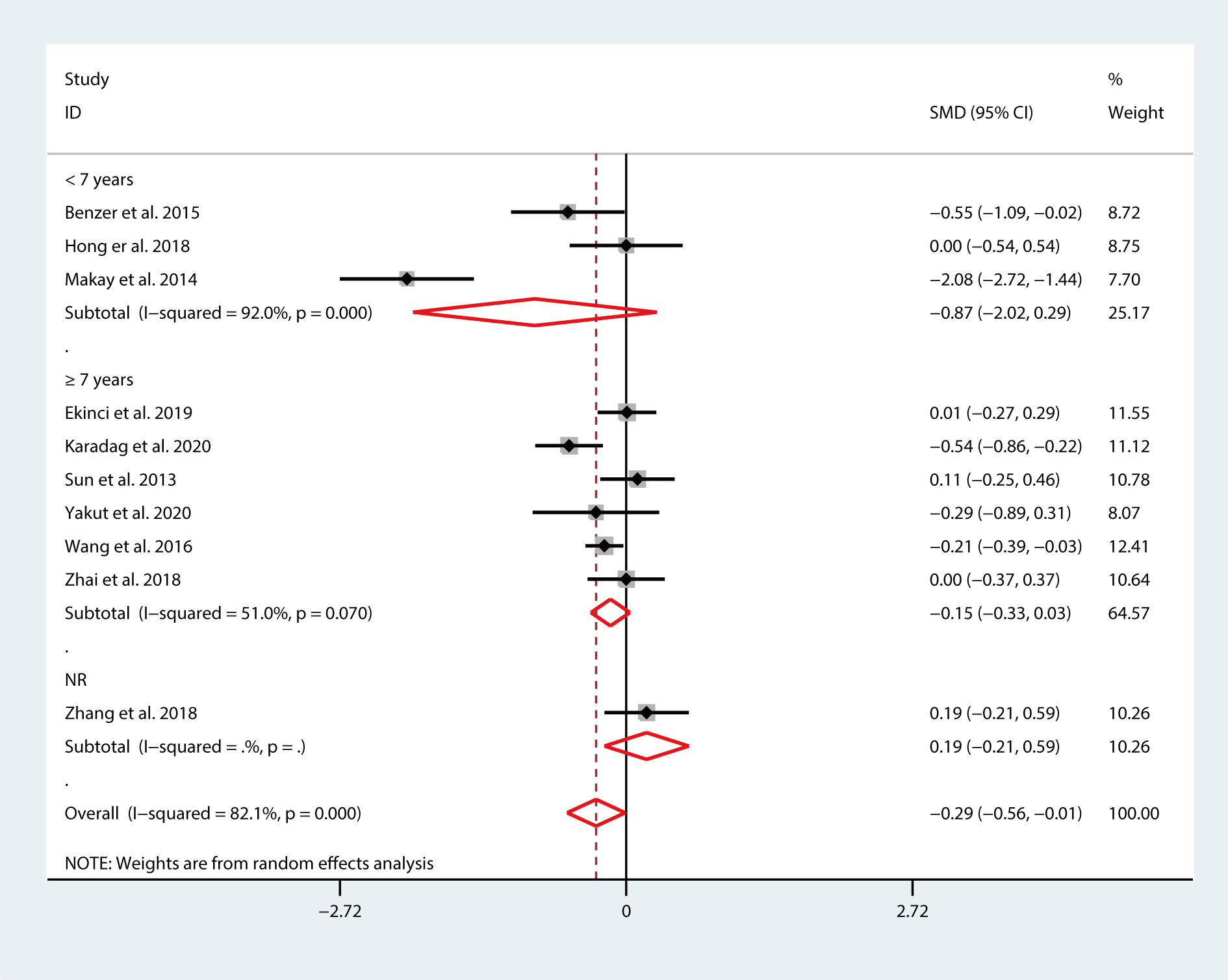

Supplement: Supplemental Material [file KBIE_A_1865607_SM3228.zip › supplement/Supplement 6.tif]

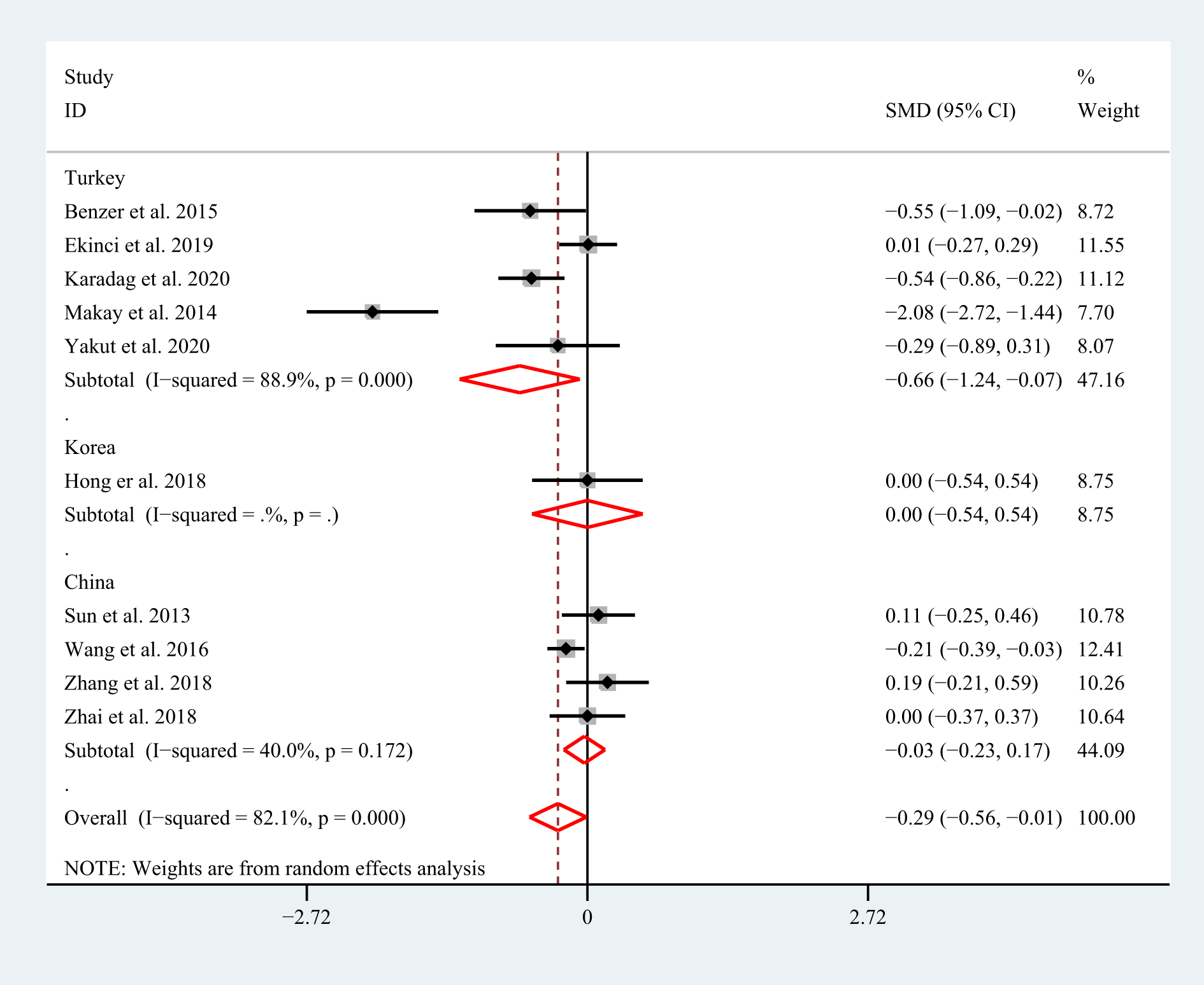

Supplement: Supplemental Material [file KBIE_A_1865607_SM3228.zip › supplement/Supplement 7.tif]

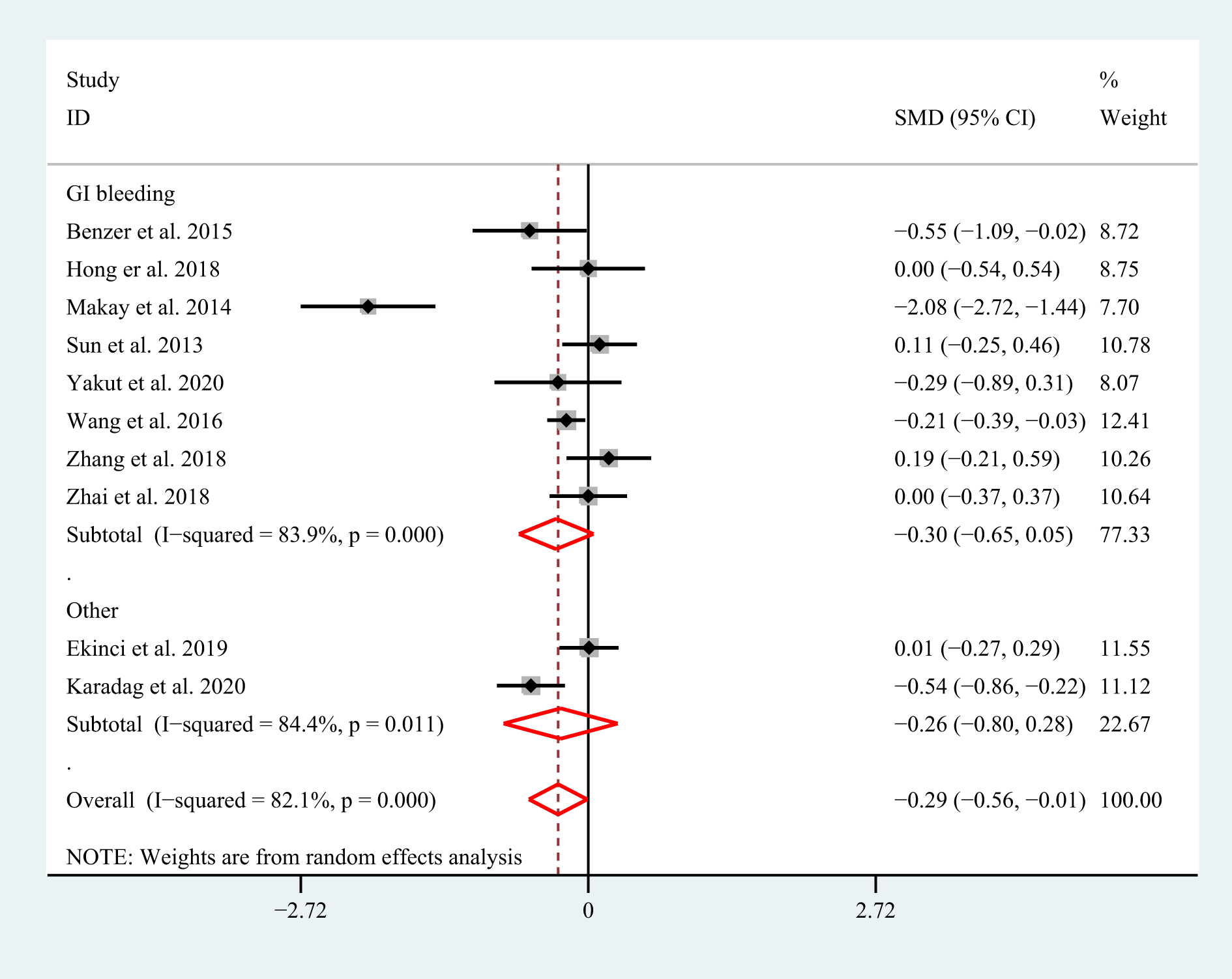

Supplement: Supplemental Material [file KBIE_A_1865607_SM3228.zip › supplement/Supplement 8.tif]

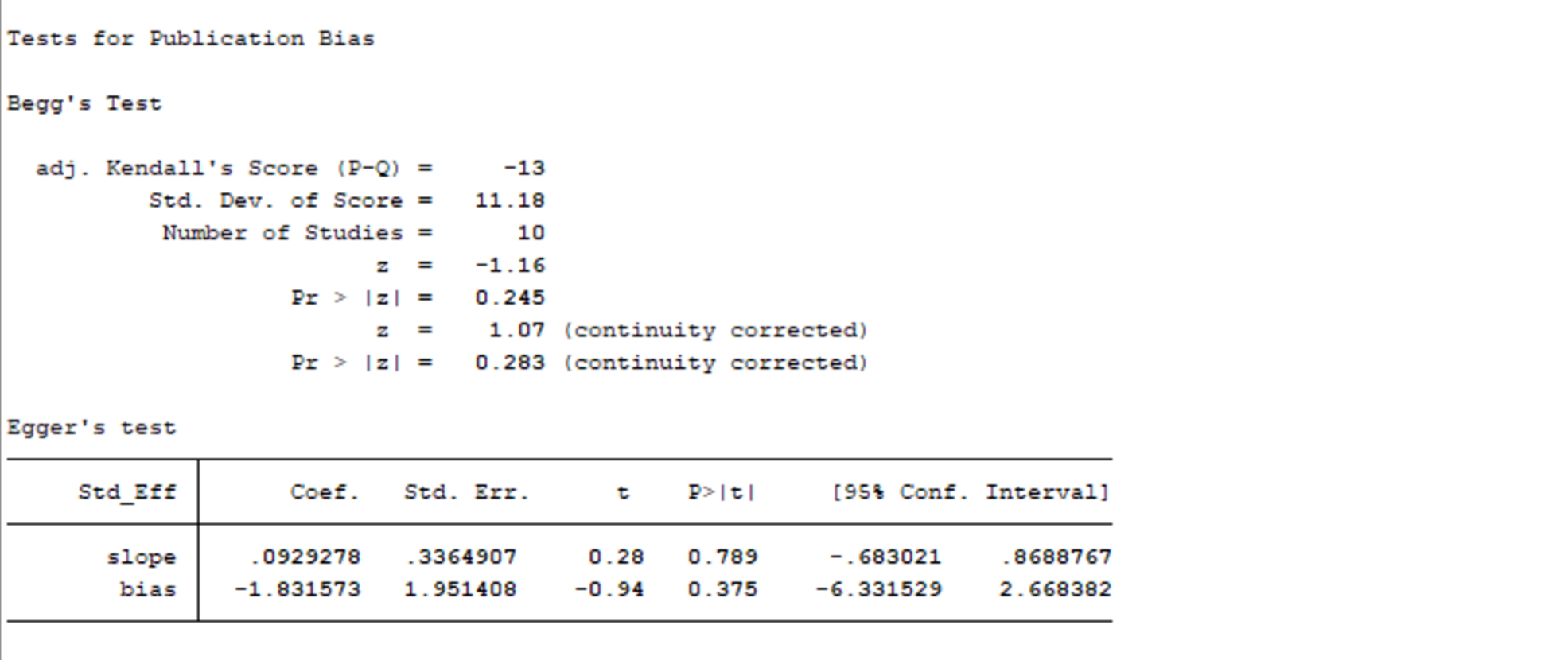

Supplement: Supplemental Material [file KBIE_A_1865607_SM3228.zip › supplement/Supplement 9.tif]
